# A case report of double Meckel’s diverticulum with a mobile cecum

**DOI:** 10.1186/s12893-026-03694-6

**Published:** 2026-04-02

**Authors:** Kenta Okumura, Kie Matsumoto, Yuka Masuki, Sangyo Kashima, Genta Nagakubo, Yusuke Seki, Shuntaro Yoshimura, Yoshiharu Kono, Jo Tashiro, Hidenori Urabe, Shigeki Morita, Kazuhiko Mori

**Affiliations:** 1 Department of Digestive Surgery, Mitsui Memorial Hospital, 1 Kanda-Izumi-Cho, Chiyoda-Ku, Tokyo, 101-8643 Japan; 2 Hepato-Biliary-Pancreatic Surgery Division, Department of Surgery, Graduate School of Medicine, The University of Tokyo, 7-3-1 Hongo, Bunkyo-Ku, Tokyo, 113-0033 Japan; 3 Department of Pathology, Mitsui Memorial Hospital, 1 Kanda-Izumi-Cho, Chiyoda-Ku, Tokyo, 101-8643 Japan

**Keywords:** Meckel’s diverticulum, Double MD, Mobile cecum, Gastrointestinal hemorrhage, Laparoscopy-assisted surgery, Diverticulectomy, Wedge resection, Case report

## Abstract

**Background:**

Meckel’s diverticulum is the most prevalent congenital malformation of the gastrointestinal tract. However, the coexistence of double Meckel’s diverticula and a mobile cecum is rare and has rarely been reported in the literature.

**Case presentation:**

A 40-year-old male presented with melena, and his condition subsequently deteriorated, with a blood pressure of 73/57 mmHg and a heart rate of 56 beats/min. Contrast-enhanced computed tomography revealed a giant blind lumen filled with hematoma and malposition of the right colon. The patient underwent an emergency surgery. Although a smaller diverticulum was initially found via minilaparotomy, the discrepancy with preoperative imaging prompted further laparoscopic exploration, which revealed a second large diverticulum measuring 170 mm. Both diverticula were resected. Pathological examination revealed that the smaller diverticulum contained ectopic duodenal and gastric mucosa, whereas the larger diverticulum contained ectopic gastric mucosa and pancreatic tissue.

**Conclusions:**

The coexistence of double Meckel’s diverticula and a mobile cecum is a rare anomaly. Although life-threatening hemorrhage from a Meckel's diverticulum is an uncommon presentation in adults, this case highlights the critical need to thoroughly explore for additional lesions when intraoperative findings do not fully align with preoperative imaging. Furthermore, surgical strategies must be tailored to the specific morphology of each diverticulum.

## Background

True diverticula of the small intestine are congenital disorders that involve the same layers as the adjacent intestinal walls (mucosa, submucosa, muscularis, and serosa) [1]. These include Meckel’s diverticulum (MD) and intestinal duplication cysts (IDCs). MD results from the remnant vitelline duct in the embryo and is a relatively common diverticulum that is present in approximately 2% of autopsies [2]. In contrast, the incidence of IDC is 1:4,500 births, accounting for 0.2% of all children [3]. MD is typically found 15–120 cm proximal to the ileocecal valve [1]. In most cases, MD is asymptomatic and is diagnosed incidentally using computed tomography or is detected during abdominal surgery.

The clinical presentation varies significantly according to age. In the pediatric population, gastrointestinal hemorrhage is the most common complication, accounting for approximately 50% of cases [2]. Conversely, intestinal obstruction is the leading cause of symptoms in adults, accounting for 35.6% of complications, whereas hemorrhage is relatively rare [1]. Other complications include intussusception, diverticulitis, perforation, and neoplasms [4]. Although a single MD is a known cause of gastrointestinal hemorrhage, the presence of double MDs complicates the clinical scenario. This highlights the importance of recognizing that identifying one diverticulum does not eliminate the possibility of another bleeding source.

A mobile cecum is also a congenital malformation. Wolfer et al. [5] reported that 10% of the population has incomplete peritoneal fixation of the ascending colon and cecum. Autopsy studies have revealed an 11% incidence of freely mobile right colon and a 26% incidence of cecal mobility sufficient to cause symptoms [6]. Mobile right colon syndrome is a relatively rare congenital abnormality that presents as intestinal obstruction during childhood [7, 8]. This syndrome primarily affects children, and adult-onset cases are relatively rare [7, 8]. It is characterized by chronic right lower abdominal pain in the absence of appendicitis or other pathological findings during surgery [9].

We encountered an adult case of double Meckel’s diverticula and a mobile cecum presenting with massive hemorrhage. This case serves as a crucial reminder that Meckel's diverticulum in adults can cause catastrophic, life-threatening bleeding. In this report, we propose a hypothesis concerning the causal link between a large diverticulum and a mobile cecum.

## Case presentation

**Fig. 1:**
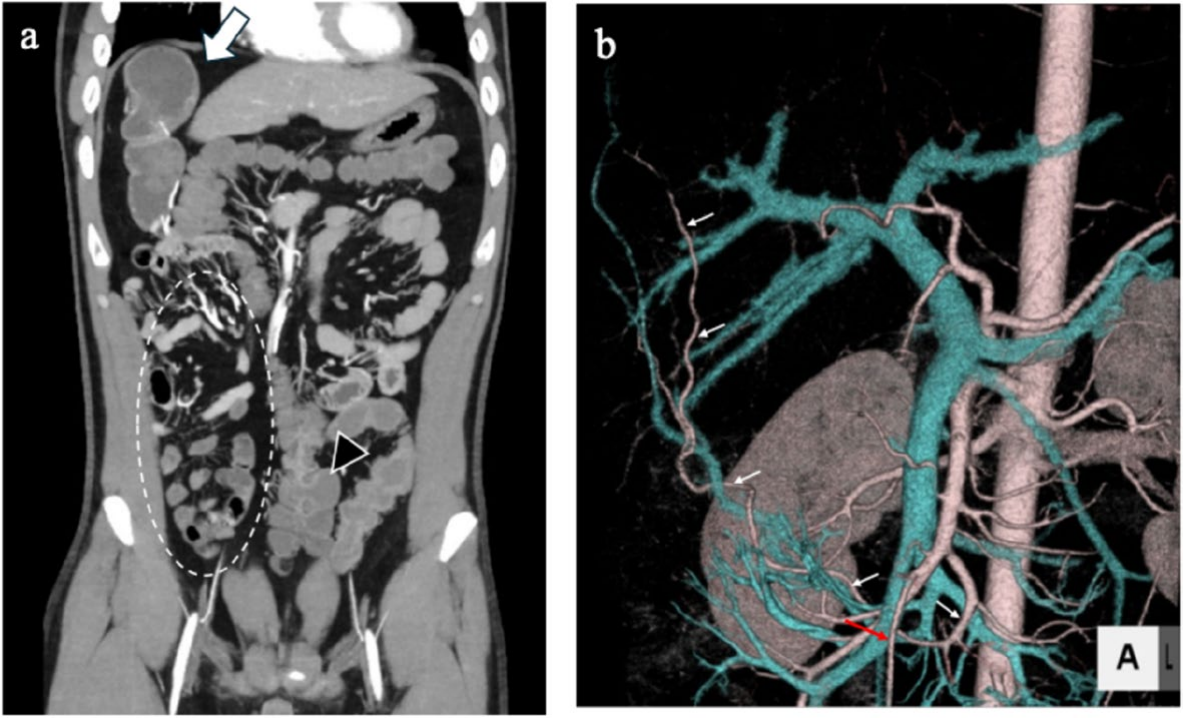
Preoperative computed tomography (CT) images. **a** Coronal CT image showing a 145-mm diverticulum under the right diaphragm with a hematoma inside (white block arrow). The ascending colon (black arrowhead) is located near the midline, with small intestinal loops (inside the white dashed circle) on the right lateral side. **b** Reconstructed vascular image of the superior mesenteric artery. The artery feeding the diverticulum (white arrows) originates from the superior mesenteric artery and courses behind the ileocecal artery (red arrows)

**Fig. 2:**
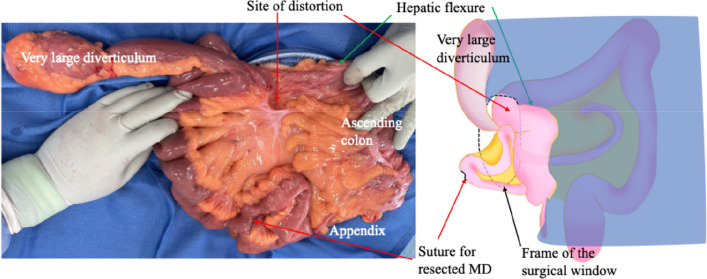
Intraoperative findings (after resecting the smaller diverticulum). A very large diverticulum was identified and removed from the peritoneal cavity. As predicted by preoperative CT, the ascending colon was volvulated anteriorly, and a considerable length of the intestinal loop was displaced laterally, passing behind the ascending colon. Before this surgical view was established, a 60-mm diverticulum located on the antimesenteric side was resected. The very large diverticulum was also located on the antimesenteric side, and its base was very close to the distorted site of the colon

**Fig. 3:**
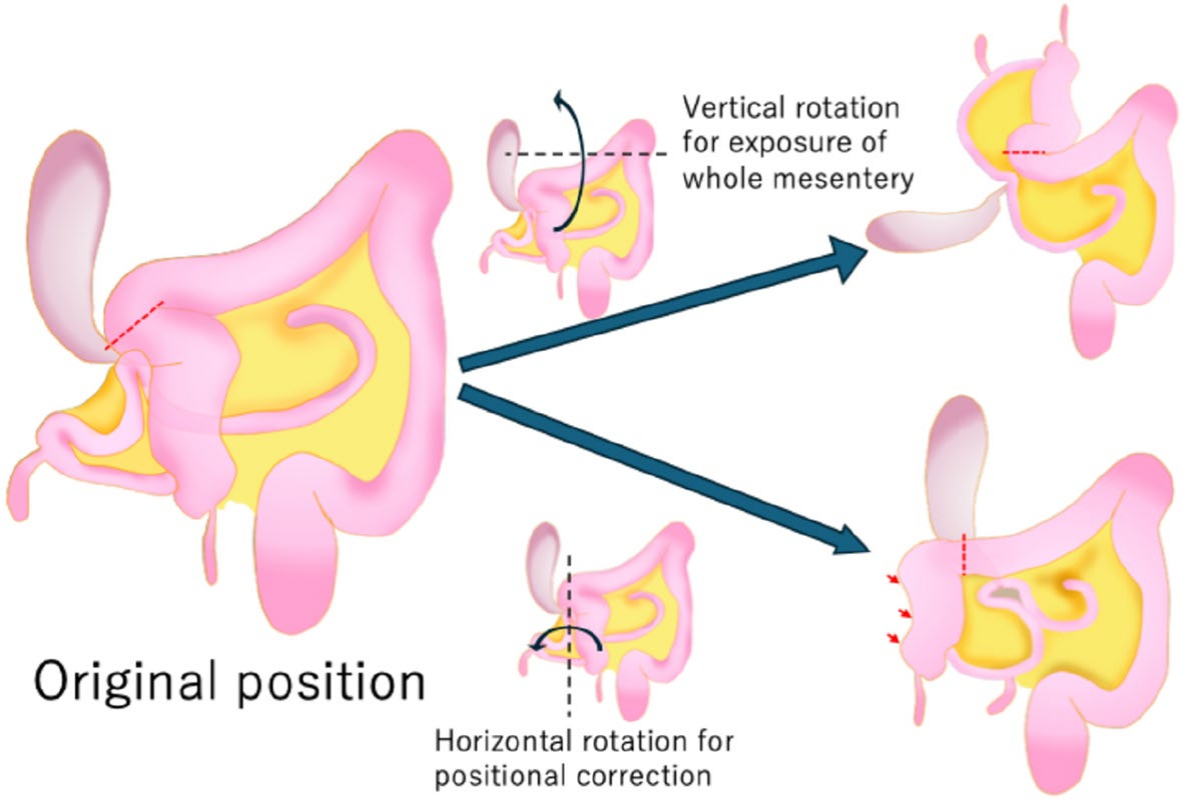
Schematic of intestinal loops and diverticula. Schema of the intestinal loops and diverticula in their original positions and rotation maneuvers required for either exposure of the whole mesentery or positional correction. The mobile cecum was accompanied by total fixation failure of the right colon. The right colon was rotated horizontally and shifted toward the midline, allowing a long, small intestinal loop, including two diverticula, to migrate to the right side by passing behind the ascending colon. The dashed red line indicates the distortion site in the transverse colon. Vertical rotation of the right colon from its original position facilitated the exposure of the entire small intestinal mesentery and resection of the very large diverticulum. Horizontal rotation was required to correct the positions of the large and small intestines. After diverticular resection, the ascending colon was fixed to the right side of the peritoneal cavity using interrupted sutures (red arrows)

**Fig. 4:**
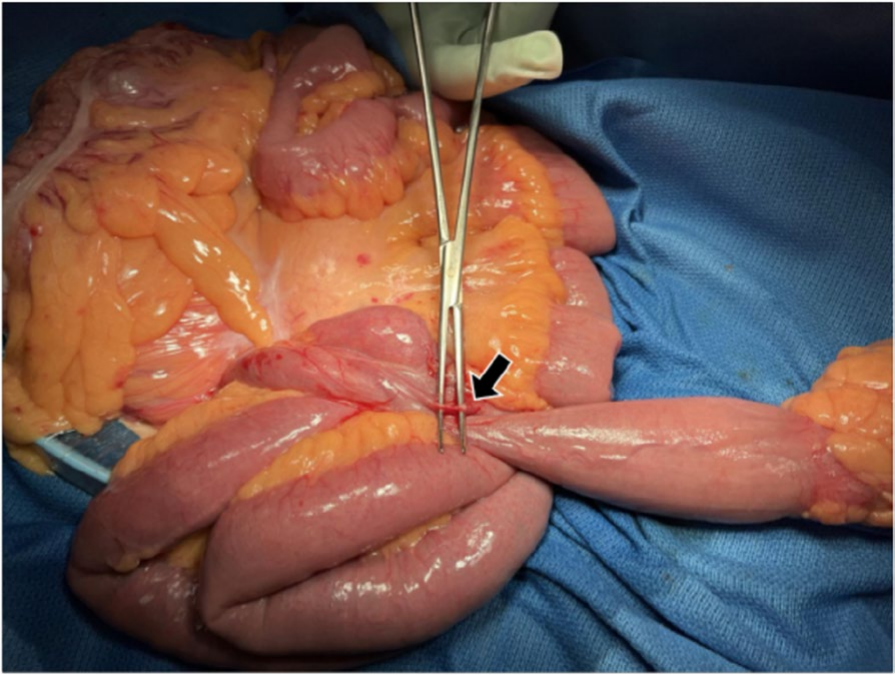
A thick feeding artery of the very large diverticulum thick pulsating artery distributed in the diverticulum was noted in the subserosal layer of the intestinal loop adjacent to the base of the diverticulum. The artery was isolated using a Kelly clamp before division (black arrow)

**Fig.5:**
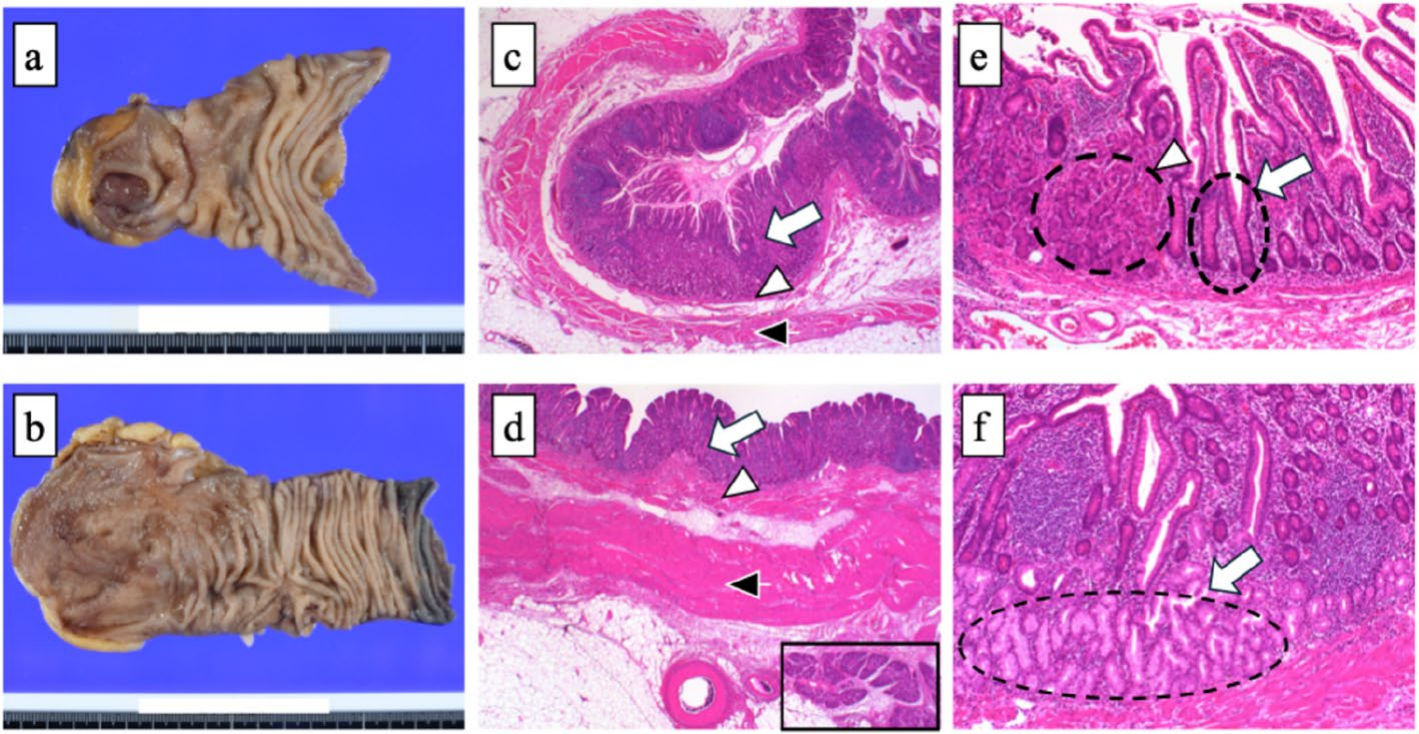
Histological examination of the resected double diverticula. **a** Resected sample. The anal diverticulum measured 85 × 60 mm. **b** Resected sample. The oral diverticulum measured 170 mm × 75 mm. **c**, **d** (hematoxylin and eosin (HE) staining × 20) Pathological findings of the anal (**c**) and oral (**d**) diverticula. True diverticula were diagnosed on the basis of the presence of mucosa (white arrow), muscularis mucosa (white arrowhead), muscularis propria (black arrowhead), and serosa underneath. The oral diverticulum had subserosal pancreatic acini and ducts (black boxes). **e** (HE stain × 200) Pathological findings of the anal diverticulum. The small intestinal mucosa (white arrow) and gastric mucosa with fundic glands (white arrowhead) are shown. **f** (HE stain × 200) Pathological findings of the oral diverticulum. The pyloric glands deep in the mucosal lamina propria (white arrow) are visible

A 40-year-old male presented with black stools noted several days prior. The patient had no significant medical or surgical history. His vital signs included a body temperature of 36.9 °C, a blood pressure of 120/72 mmHg, a heart rate of 90 bpm, a respiratory rate of 22/min, and an SpO2 of 98% (room air). Blood tests revealed severe anemia (Hb 8.5 g/dL). Upon admission, an initial conservative treatment consisting of fasting, fluid resuscitation, and blood transfusion was performed. Endoscopic examinations were planned on a standby basis. However, on the second day of hospitalization, the patient developed massive amounts of dark red stools. His blood pressure decreased to 73/57 mmHg, with a heart rate of 56 bpm. Contrast-enhanced computed tomography (CT) revealed a large diverticulum measuring 145 mm in the right subdiaphragmatic region with an intraluminal hematoma and malposition of the ascending colon. No contrast-enhanced extravasation was observed. The feeding artery leading to the diverticulum was identified as originating from and passing behind the superior mesenteric artery, and the ascending colon was not fixed to the retroperitoneum but was displaced in the midline (Fig. 1). Emergency surgery was performed under the diagnosis of a life-threatening hemorrhagic diverticulum. We started with a 6-cm upper right pararectal incision (minilaparotomy) for exploration, and the intestinal loop, including the ileocecal region, was exteriorized and examined. The ascending colon was not fixed to the retroperitoneum or the ileocecal region, and the proximal transverse colon was easily mobilized extracorporeally (mobile cecum). A 60-mm diverticulum located 60 cm proximal to the Bauhin valve was identified. The diverticulum was excised via wedge resection because of its short length, and the bowel wall was closed using Gambee sutures (Fig. 2). However, this resected specimen was significantly smaller than the large diverticulum seen on preoperative CT. Suspecting a second lesion, we performed laparoscopic exploration using an Alexis O device (Applied Medical, Tokyo, Japan) for minilaparotomy and additional ports. Additional ports were placed in the left lateral and upper mid-abdominal regions. This revealed a second diverticulum measuring 170 mm, located 130 cm proximal to the Bauhin valve (Fig. 2). This very large diverticulum was located mainly behind the mesocolon and was aimed cranially, with its apex adhering to the hepatic dome of the diaphragm. The laparoscopic procedure was abandoned, and the entire length of the diverticulum could be outlined via a 6 cm wound. However, the base of the large diverticulum was fixed to the retroperitoneum and poorly visualized. Further trials were conducted to correct the position of the right colon and to identify the origin of the large diverticulum (Fig. 3). The locus of the transverse colon near the hepatic flexure (dashed red line in Fig. 3) was observed between the mobile and fixed portions of the right colon. The position of the right colon was corrected by horizontal rotation, and the entire mesentery of the small intestine and its relation to the large diverticulum were exposed by vertical rotation of the right colon. After the aforementioned maneuvers were performed, the large diverticulum was exteriorized through additional adhesiolysis. Pulsation of a thick feeding artery was visible under the serosa of the adjacent intestinal wall near the base of the diverticulum and was isolated using a Kelly clamp (Fig. 4). Considering that the large diverticulum was vertically long, we chose to perform simple diverticular resection using a linear stapler. The mobile cecum was fixed using sutures in the right lower quadrant, and the position of the migrating intestines was corrected. The patient had persistent melena until postoperative Day 4, requiring cautious reinitiation of oral intake. The patient was discharged on the 9th postoperative day without complications. Although the pathological study revealed no histological evidence of hemorrhagic lesions within the two diverticula, we concluded that the massive gastrointestinal bleeding was derived from the larger diverticulum on the basis of the preoperative CT and macroscopic findings of the retrieved specimens. Histological examination of the resected diverticula revealed that the distal diverticulum contained ectopic gastric and duodenal mucosa. The proximal diverticulum contained ectopic gastric mucosa and pancreatic tissue (Fig. 5). The resection margins of both specimens were negative for ectopic mucosa. Given that both were located on the antimesenteric side, the two diverticula were diagnosed as double MDs.

## Discussion

The patient presented here developed two coincident developmental disorders of the intestine: double MDs and a mobile cecum. In this case, the clinical manifestation of massive gastrointestinal hemorrhage requiring emergent surgery was derived from a large MD. In addition, the reported case had two diverticula in the ileum, both of which were pathologically diagnosed as true diverticula and comprised all the layers of the intestinal wall. Two distinct types of true diverticula, MD and IDC, are known, but they are histologically indistinguishable. Multiple MDs are extremely rare because it is generally believed that an MD is a remnant of the yolk intestinal tract and that only one can exist. However, in this case, the two diverticula were diagnosed as MDs on the basis of the following three features: 1) antimesenteric location, 2) lack of a proper mesentery, and 3) a separate blood supply from the subserosal layer. Unlike MDs, IDCs are located on the mesenteric side and share a blood supply with the adjacent intestine. This distinction is crucial for the diagnosis.

Symptomatic MD in adults is uncommon and, compared with the pediatric population, gastrointestinal bleeding is an atypical presentation, making preoperative diagnosis challenging [1]. Generally, identifying MD on CT is difficult because it is often indistinguishable from normal small-bowel loops. 99mTc scintigraphy and double-balloon endoscopy have been used to confirm the diagnosis of MD; however, in the present case, the diagnosis was easily established because of the exceptionally large size of the diverticulum and the presence of a high-density hematoma within the lumen.

The presence of two MDs is extremely rare. Losanoff hypothesized that overlapping segments of the vitelline duct during embryogenesis could explain the formation of double MDs [10]. To date, a total of 21 cases of double MDs have been reported in the literature [10–30]. Our review of these cases revealed a slight male preponderance, which is consistent with the general trend observed in single MD [2], although the sample size is limited. Although single MDs are typically short, with an average length of approximately 3 cm [1], the diverticula in our case were exceptionally large, measuring 170 mm and 60 mm. Their size was remarkable, even compared with that of previously reported double MD cases. A large MD is associated with various complications, such as bowel obstruction [31–33]. Despite its large size, the patient did not have an episode of bowel obstruction, and the first clinical manifestation was a life-threatening hemorrhage in his 5th decade of life.

We employed two resection techniques tailored to the characteristics of each diverticulum. For smaller lesions, wedge resection was performed because a short diverticulum with a length-to-diameter ratio < 2:1 is associated with a greater risk of ectopic mucosa at the base [34, 35]. In contrast, for a large diverticulum with a ratio greater than 2:1, simple diverticulectomy after ligation of the large feeding artery is preferred. The absence of ectopic mucosa at the resection margins in both specimens supports the validity of this surgical strategy.

The relationship between double MDs and the mobile cecum is of particular interest. Because MD formation occurs in the 4th week of gestation [36], preceding cecal fixation in the 12th week [37], the presence of MD may have disturbed the fixation process. Furthermore, we hypothesized that the exceptionally large size of the proximal diverticulum resulted from chronic obstruction or torsion caused by the mobile cecum, leading to secondary hypertrophy. However, this mechanism remains speculative, as it is based on a single case.

## Conclusions

The coexistence of double MDs and a mobile cecum is very rare. Furthermore, life-threatening gastrointestinal bleeding from a Meckel's diverticulum is an exceptionally uncommon clinical presentation in adults. This case highlights the critical need for surgeons to maintain a high index of suspicion for multiple anomalies. Identifying a single diverticulum should not preclude a thorough search for additional lesions, especially when intraoperative findings do not completely align with preoperative imaging. Furthermore, tailoring the surgical approach, such as using wedge resection for short diverticula and simple diverticulectomy for large diverticula, ensures both optimal hemorrhage control and the complete removal of ectopic mucosa.

## Data Availability

No datasets were generated or analysed during the current study.

## Abbreviations

CT: Computed tomography
HE: Hematoxylin and eosin
IDC: Intestinal duplication cyst
MD: Meckel’s diverticulum
